# Variation in developmental patterns among elite wheat lines and relationships with yield, yield components and spike fertility

**DOI:** 10.1016/j.fcr.2016.07.019

**Published:** 2016-09

**Authors:** Oscar E. Gonzalez-Navarro, Simon Griffiths, Gemma Molero, Matthew P. Reynolds, Gustavo A. Slafer

**Affiliations:** a Crop Genetics Department, John Innes Centre, Norwich Research Park, Norwich, Norfolk NR4 7UH, UK; b CIMMYT (International Maize and Wheat Improvement Center), Apdo Postal 6-641, 06600 Mexico D.F., Mexico; c AGROTECNIO (Center for Research in Agrotechnology), and Department of Crop and Forest Sciences, University of Lleida, Av. Rovira Roure 191, 25198 Lleida, Spain; d ICREA, Pg. Lluís Companys 23, 08010 Barcelona, Spain

**Keywords:** Fruiting efficiency, Stem elongation, Spike fertility, Grain number, *Triticum aestivum* L.

## Abstract

Developmental patterns strongly influence spike fertility and grain number, which are primarily determined during the stem elongation period (i.e. time between terminal spikelet phase and anthesis). It has been proposed that the length of the stem elongation phase may, to an extent, affect grain number; thus it would be beneficial to identify genetic variation for the duration of this phase in elite germplasm. Variation in these developmental patterns was studied using 27 elite wheat lines in four experiments across three growing seasons. The results showed that the length of the stem elongation phase was (i) only slightly related to the period from seedling emergence to terminal spikelet, and (ii) more relevant than it for determining time to anthesis. Thus, phenological phases were largely independent and any particular time to anthesis may be reached with different combinations of component phases. Yield components were largely explained by fruiting efficiency of the elite lines used: the relationships were strongly positive and strongly negative with grain number and with grain weight, respectively. Although fruiting efficiency showed a positive trend with the duration of stem elongation that was not significant, a boundary function (which was highly significant) suggests that the length of this phase may impose an upper threshold for fruiting efficiency and grain number, and that maximum values of fruiting efficiency may require a relatively long stem elongation phase.

## Highlights

• Time to terminal spikelet and from then to anthesis were largely independent.
• The length of the stem elongation phase was slightly but positively related to grains per m^2^.
• Fruiting efficiency was critical for determining grain number, but it was also negatively related to grain weight.
• The length of the stem elongation phase seems to have imposed an upper threshold for fruiting efficiency.

## 1 Introduction

A substantial increase in wheat yield potential is required in the coming decades, but rates of genetic gain are currently well below the level required to match the projected cereal demand (Reynolds et al., 2012, Hall and Richards, 2013, Fischer et al., 2014). Quantifying the degree of genetic variation within elite germplasm in traits which may contribute to increased yield potential is critical to the design of strategic crosses (Slafer, 2003, Foulkes et al., 2011, Reynolds et al., 2012).

Yield can be analysed in terms of the number of grains and their average weight. The capacity of the canopy to provide assimilates to fill the grains does not appear to limit grain growth in a wide range of background growing conditions and genotypes (Borrás et al., 2004, Serrago et al., 2013), even within elite high-yielding material (Pedro et al., 2011, González et al., 2014, Sanchez-Bragado et al., 2014). As grain number is strongly source-limited and highly responsive to changes in availability of assimilates (see below), grain number is more plastic than grain weight (Peltonen-Sainio et al., 2007, Sadras, 2007, Sadras and Slafer, 2012) and yield is far more commonly related to grain number than to the average weight of grains (Fischer, 2011, Slafer et al., 2014). Thus to achieve relevant genetic gains in yield potential it is important to identify traits responsible for the determination of grain number (Slafer et al., 2014).

Grain number in wheat is largely determined during the stem elongation phase (Fischer, 1985, Slafer and Rawson, 1994), when the juvenile spikes grow whilst floret developmental processes determine the survival of floret primordia (Kirby, 1988). As wheat is a cleistogamous plant, most fertile florets become grains and therefore the process of floret survival and spike growth before anthesis is critical for determining grain number (González et al., 2011a, Ferrante et al., 2013). This underlies the widely reported positive relationship between grain number and spike dry weight at anthesis, first proposed by Fischer (1985) and later validated in a wide range of cases (Slafer et al., 2005 and references quoted therein), irrespective of whether variations are produced by manipulations in growing conditions (Fischer, 1985, Savin and Slafer, 1991, Fischer, 1993, Abbate et al., 1995, Demotes-Mainard and Jeuffroy, 2004, Prystupa et al., 2004, Acreche and Slafer, 2011, Ferrante et al., 2012, Marti and Slafer, 2014) or genetically through altering dry matter partitioning to the spikes (*e.g.* Siddique et al., 1989, Slafer and Andrade, 1993, Miralles and Slafer, 1995, Flintham et al., 1997, Miralles et al., 1998, Reynolds et al., 2001, Reynolds et al., 2005) during stem elongation.

Therefore, it has been proposed that the duration of the late reproductive phase, from the initiation of terminal spikelet to anthesis (Slafer, 2012), may influence the number of grains produced by the crop. The rationale behind this proposition is that a longer phase when florets are developing may influence the likelihood of floret primordia become fertile florets, and then to set a grain (Miralles and Slafer, 2007). Empirical support to this proposition has been provided through manipulating the duration of the late reproductive phase (through changing photoperiod conditions during stem elongation) producing parallel changes in duration of the late reproductive phase and grain number (Miralles et al., 2000, González et al., 2003, González et al., 2005a, Serrago et al., 2008). This in turn may be due to two alternative, non-exclusive mechanisms: a longer period of stem elongation could (i) bring about increases in accumulated growth enhancing the resource availability for the juvenile spike in which florets are developing (the increase in fertility would then be associated with increased spike dry weight at anthesis), or (ii) allow floret primordia which would not, normally, progress to produce a fertile floret a longer period of development and eventually to be able to reach the stage of fertile floret (and then the increase in fertility would be associated with increases in fruiting efficiency; the efficiency with which resources allocated to the spike by anthesis are used to set grains).

As time to anthesis is critical for crop adaptation (Richards, 1991, Worland, 1996, Slafer et al., 2015) modern, high-yielding wheat have a flowering time that has been largely optimised in most regions. Thus, optimizing the developmental pattern through changing the partitioning of developmental time to anthesis into different duration of phases occurring earlier or later than the initiation of the terminal spikelet may contribute to increasing spike fertility (Slafer et al., 2001, Miralles and Slafer, 2007, Foulkes et al., 2011, Reynolds et al., 2012). The ability of breeders to increase the duration of the late reproductive phase depends on genetic variation for this trait.

It has been shown that the duration of the different pre-anthesis phases may be independent (Whitechurch et al., 2007, Borràs et al., 2009, García et al., 2014, González et al., 2014), which is consistent with the fact that different phases vary in sensitivity to vernalisation, photoperiod, and temperature (Slafer and Rawson, 1994, Slafer and Rawson, 1995, Slafer and Rawson, 1996, Miralles and Richards, 2000, González et al., 2002). The existence of genetic variation is key to the design of strategic crosses. As breeders combine favourable alleles in order to achieve genetic progress for yield (and other complex traits), they are enthusiastic to consider potential parents from a selected group of genotypes that can be considered elite. CIMMYT has gathered a special population for studying opportunities for improvements in photosynthesis and biomass simultaneously while maintaining high levels of harvest index, namely the CIMMYT Mexico Core Germplasm (CIMCOG). It includes advanced hexaploid wheat lines that have the potential to bring together traits required for producing step changes in yield gains, as well as historical cultivars and high-yielding durum wheats for reference. CIMCOG was the focal panel used by the Wheat Yield Consortium to studying alternatives for further raising yield potential (Reynolds et al., 2011).

The objective of the present study was to determine the degree of variation in patterns of phenological development within the elite germplasm of the CIMCOG population, ascertaining whether the differences were related to traits determining spike fertility within the population.

## 2 Materials and methods

Four field experiments were conducted at the Mexican Phenotyping Platform (MEXPLAT) established at the research station “Centro Experimental Norman E. Borlaug” (CENEB), near Ciudad Obregon, Sonora, Mexico (27°33′N, 109°09′W, 38 masl), with conditions that represent the high-yield potential wheat mega-environment 1 (Braun et al., 2010). The soil was a Chromic Haplotorret (Vertisol Calcaric Chromic), low in organic matter (<1%), and slightly alkaline (pH = 7.7).

### 2.1 Plot information

Experiments 1 and 2, differing in the sowing system, were conducted in 2010/11, experiment 3 in 2011/12, and experiment 4 in 2012/13. Plots in experiments 1, 3, and 4 were carried out in raised beds while experiment 2 had flat (conventional) plots, and in all cases plots were large (17.7–30 m^2^) and sown within the optimal period in the region and with optimal sowing densities (Table 1).

All plots were grown under optimal conditions: they were fertilised and irrigated to avoid N and water stress, and biotic stresses were prevented or controlled (weeds were removed by hand throughout the growing season and diseases and insects prevented by applying recommended fungicides and insecticides at the doses suggested by their manufacturers).

### 2.2 Treatments

The treatments analysed in this study consisted of a subset of the CIMCOG panel of 27 genotypes (comprised of 22 elite lines, 4 historic lines, and 1 *T. durum*) that were grown throughout the 4 field experiments. The original CIMCOG panel of 60 genotypes was only grown and measured in experiments 1 and 2. In experiments 3 and 4 the subset of 27 lines were selected to represent fairly the complete panel (based on results of the first two experiments). All four experiments were designed in randomized complete blocks with two replicates on experiment 2 and three replicates on experiments 1, 3, and 4.

### 2.3 Determination of key phenology stages

Plots were inspected periodically after sowing. Seedling emergence was determined when half of the seedlings in the plot reached the point when the tip of the first leaf emerged from the coleoptile. From then on, one plant per plot (two or three per genotype depending on each experiment) was sampled (once a fortnight at the beginning and then increasing the frequency as the plot was approaching terminal spikelet initiation, around late January, to up to three times a week) and dissected under a binocular microscope (Carl Zeiss, Germany) to record the stage of development of the apex and so determine the timing of initiation of the terminal spikelet with accuracy. Thereafter the plots were regularly inspected to determine the timing of anthesis when half of the spikes of the plot had anthers extruded.

### 2.4 Sampling and determinations

A sample of 0.5 m of two rows was taken seven days after anthesis, in which above-ground biomass was determined dividing it into spikes and the rest of the canopy. In experiment 4, a sub-sample was taken in which all of the immature grains were removed from the spikes so that the non-grain spike dry weight at a week after anthesis could be obtained. The elimination of the weight of the grains is relevant as they may represent a sizeable, and genotypically variable, portion of the spike dry weight at that stage (7d after anthesis). Their inclusion would overestimate the bulk of resources that were available for the set grains(Fischer, 2011 and references therein). With these values we estimated the proportion of grain and non-grain spike dry weight at a week after anthesis for each genotype to estimate the non-grain spike dry weight in all previous experiments. The reported spike dry weight at anthesis is the value of spike dry weight 7 days after anthesis multiplied by each genotypic factor obtained from experiment 4. The rate of grain filling was determined by calculating a linear model for the relationship between time from anthesis to maturity (grain filling period) and grain weight.

At maturity, yield was determined from harvesting the plot (excluding the extreme 50 cm to avoid border effects) using standard protocols (Pask et al., 2012). Before that, 100 fertile culms were sampled, dried, weighed and threshed to allow calculation of yield components.

With the measurements of grain number at maturity and non-grain spike dry weight one week after anthesis we estimated fruiting efficiency; i.e. the efficiency by which dry matter allocated to the spikes at anthesis is used to determine the survival of floret primordia and set grains (Ferrante et al., 2012, García et al., 2014).

### 2.5 Analyses

Analyses of variance (ANOVA) and of principal components (PCA) were performed using R 3.0.2 (R Development Core Team). PCA was plotted with the ggbiplot package from R. Regression analysis was conducted to establish the correlation between traits, and figures were produced, using GraphPad Prism 5 (2007). For the relationship between fruiting efficiency and duration of the phase from terminal spikelet to anthesis we also fitted a boundary function for establishing an upper threshold (a line edging the upper limit of the data-cloud; Casanova et al., 1999) describing the highest fruiting efficiencies observed over the range of durations of this phase measured; a procedure commonly used to establish upper limits in ecology (e.g. Cade and Noon, 2003) and agronomy (e.g. Sadras and Angus, 2006). To derive the boundary function we subdivided the phase duration data in intervals of 2 days (from 36 to 48 d) and fitted a regression considering the maximum values of fruiting efficiency within each interval.

## 3 Results

### 3.1 Representativeness of the subset

**Fig. 1:**
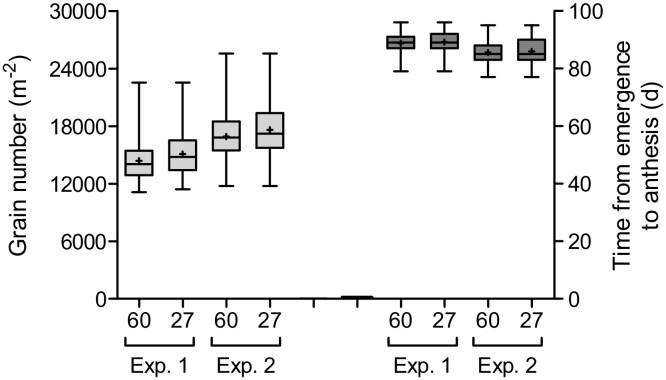
Boxplot of grain number (left side) and days to anthesis (right side) considering either the complete CIMCOG panel of 60 lines (60) or its subset of 27 lines grown throughout the four experiments (27) for each of the two experiments in which they were grown.

The 27 lines selected to represent the CIMCOG population in the 4 studies were shown to be representative of the whole population. The duration from seedling emergence to anthesis and the number of grains per unit land area (the two most integrative traits considered in this study), for the complete CIMCOG panel (60 lines) and the subset of 27 lines studied here show similar variability (Fig. 1).

Although the genotype by environment interaction (GxE) was statistically significant in most of the traits analysed in this study, in all cases the mean squares of GxE were much smaller than those of the genotypes. For instance, the magnitude of the genotypic effects was 19-fold greater than that of the GxE for the number of grains per m^2^ (and it was 56-fold greater for the average grain weight). The genotypic effects were also much greater than the GxE interaction for the duration of the two phenological phases considered here, from sowing to terminal spikelet (5-fold greater) and from then to anthesis (4-fold greater). Finally, the two physiological determinants of grain number also showed larger mean squares for genotypes than for the GxE (Table 2). Therefore, even though the GxE interaction was statistically significant the genotypic effects can be reliably considered across environments. For simplicity in most of the Results section of this paper we showed the averages across environments for each genotype, but in the last part we offered a principal component analysis which the GxE interaction is unavoidably expressed (and correspondence of conclusions derived from both analyses reinforce the usefulness of genotypic means in this study).

In the rest of this Results section all the analyses will be shown considering both the whole subset of 27 lines representing the whole CIMCOG population as well as restricting the variability to the 22 lines of this subset which are exclusively elite hexaploid lines (disregarding the four historical cultivars and the durum wheat). Therefore, any differences in results from analysing the 27 or the 22 lines would be the influence of the historic lines and/or the tetraploid wheat (*T. durum*) in the overall analysis.

### 3.2 Phenology

**Fig. 2:**
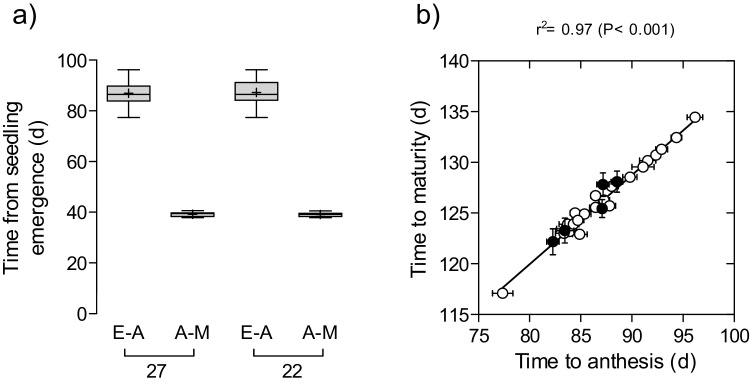
Boxplot for time from seedling emergence (E) to anthesis (A), and from then to maturity (M) considering the whole subset of 27 lines or restricting the variation to the 22 elite hexaploid lines (i.e. excluding the 4 historical and the *T. durum* cultivars) (a); and relationship between time from seedling emergence to either anthesis or maturity (b). Open circles represent the 22 elite hexaploid lines and closed circles represent the 4 historical and the *T. durum* cultivars.

The subset of 27 genotypes analysed throughout this chapter, varied noticeably in time to anthesis (Fig. 2). The variation was not due to the inclusion of the historic cultivars or due to the durum wheat cultivar, it was actually evident within the 22 lines of elite hexaploid wheat as well (Fig. 2a).

Variation in the duration of grain filling was much lower (Fig. 2a), as the time to maturity was strongly correlated with time to anthesis (Fig. 2b). In fact, the relationship between time to maturity and time to anthesis (in both cases from seedling emergence) was extremely high (r^2^ = 0.97_27lines_ and 0.98_22lines_), the slope very close to 1 (0.9 in both cases), and the intercepts (reflecting the overall average duration of grain filling) exhibited little variation (49.8 ± 2.6_27lines_ days and 49.7 ± 2.6_22lines_ days) (Fig. 2b).

**Fig. 3:**
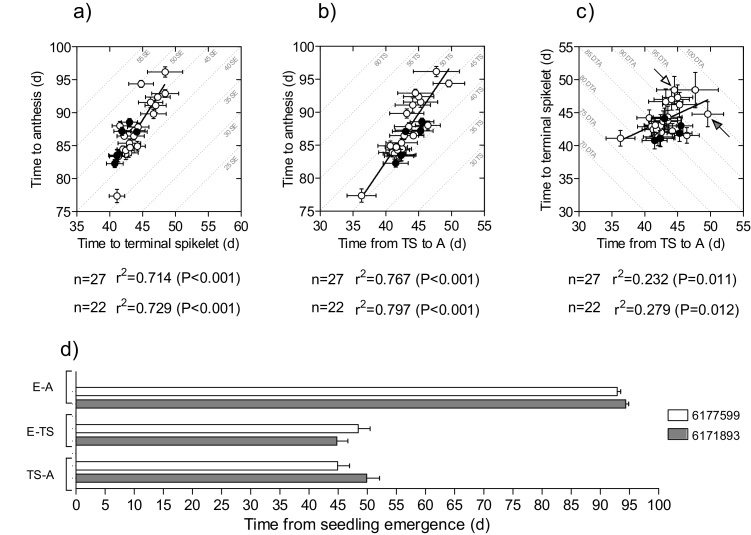
Relationships between time to anthesis and either time to terminal spikelet (a), or time from then to anthesis (b), and relationship between these two component phases (c). Within each of the panels, isolines for the same duration of complementary phases were drawn. They were (the stem elongation period (SE) in panel a, the time to terminal spikelet (TS) in panel b, and the time to anthesis (DTA) in panel c. Each data-point is the average across the 4 environments and segments stand for the standard error of the means (not seen when smaller than the size of the symbol). Open circles represent the 22 elite hexaploid lines and closed circles represent the 4 historical and the *T. durum* cultivars. The arrows in panel c point the genotypes 6177599 (open arrow head), and 6171893 (closed arrow head) illustrating a pair of genotypes with similar time to anthesis but different developmental partitioning.

In general, the variation found in phenology and the relationships between the durations of different phases were quite similar (both in terms of ranges explored and in degree of association between phases in the regressions) when analysing the whole subset of 27 lines or restricting it to 22 elite hexaploid lines disregarding the 4 historic cultivars and the *T. durum* (Fig. 3).

Time from seedling emergence to anthesis was also highly correlated with the duration of its two component phases: time from emergence to terminal spikelet (Fig. 3a) and time from terminal spikelet to anthesis (Fig. 3b). Despite the similar relationships, it seemed that the duration of the late reproductive phase was more relevant than that of the period from emergence to terminal spikelet in determining variation in total time to anthesis. This is not only because the coefficients of determination were slightly higher for the relationship with the duration of the late reproductive phase (r^2^ = 0.77–0.80) than with the time until terminal spikelet (r^2^ = 0.71–0.73), but also because the range of variation in the former (abscissa in Fig. 3b) was noticeably larger than the latter (abscissa in Fig. 3a).

More importantly, the length of either of the two phases constituting time to anthesis showed a level of independence from the other: they were significantly positively related but the proportion of the duration of time to terminal spikelet related to the duration of the late reproductive phase was only c. 25% (Fig. 3c), which indicates that cultivars may combine contrasting durations of these two phases. This shows that even within a restricted range of well adapted elite lines, there may be a large number of possible phenological combinations for reaching the same time to anthesis. For instance, a particular duration of the stem elongation phase (any of the isolines in Fig. 3a) could be combined with different durations of the phase to terminal spikelet and therefore changes in time to anthesis may be achieved by modifying exclusively the duration of phenological phases when leaf and spikelet primordia are being formed. The contrary is also true and a particular duration of the period to terminal spikelet (any of the isolines in Fig. 3b) could be combined with different durations of the late reproductive phase and therefore changes in time to anthesis may be achieved by only modifying the duration of phenological phases when floret primordia are being formed. Or a similar time to anthesis (isolines in Fig. 3c) may well be achieved combining a relatively short phase to terminal spikelet and a relatively long stem elongation phase and *vice-versa* (pairs of genotypes with the same duration to anthesis but differing in how this developmental time was partitioned between phases occurring before or after the initiation of the terminal spikelet, can easily be identified (arrowed data points in Fig. 3c and Fig. 3d).

### 3.3 Yield and yield components

**Fig. 4:**
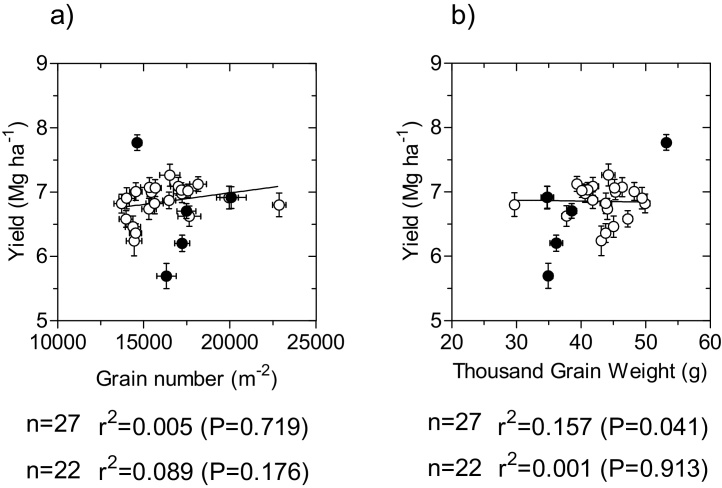
Relationships between yield and its two components: grains per unit land area (a) and the average weight of grains estimated as thousand grain weight (b). Each data-point is the average across the 4 environments and segments stand for the standard error of the means (not seen when smaller than the size of the symbol). Open circles represent the 22 elite hexaploid lines and closed circles represent the 4 historical and the *T. durum* cultivars.

Yield showed a range of more than 2 Mg ha^−1^ (from c. 5.5 to almost 8 Mg ha^−1^) when the whole subset was analysed while it was lowered to c. 1 Mg ha^−1^ when considering only the 22 elite lines (Fig. 4 on ordinates).

**Fig. 5:**
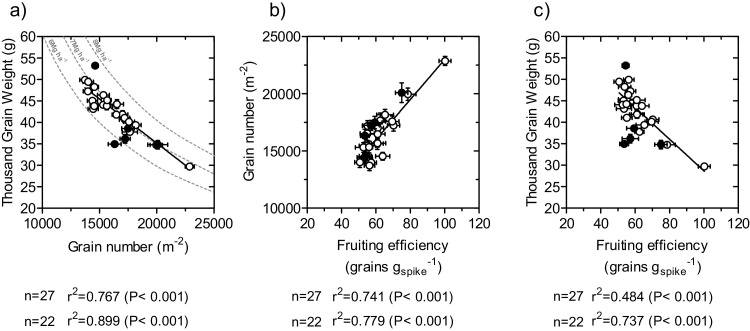
Relationships between the two major yield components (a) and between each of them (grains per unit land area [b]; average weight of grains estimated as thousand grain weight [c]) and fruiting efficiency. For panel a, isolines for the yields of 6, 7 and 8 Mg ha^−1^ were drawn. Segments stand for the standard error of the means (not seen when smaller than the sizer of the symbol). Open circles represent the 22 elite hexaploid lines and closed circles represent the 4 historical and the *T. durum* cultivars.

The difference between the consideration of the whole subset or only the 22 elite lines was noticeable in the relationships between yield and its components. For the whole subset, yield was completely unrelated to the number of grains per unit land area (Fig. 4a) and significantly related to the average weight of the grains, even though the coefficient of determination was low (Fig. 4b). However, it seems clear that the relationship was strongly dependent on two of the 27 data-points, those exhibiting the highest and the lowest yield, the former also having the highest thousand grain weight and the later having one of the lowest thousand grain weight (Fig. 4b). As these two cases correspond to the durum wheat line that produced higher yield than the hexaploid wheats and to one of the historic cultivars; when restricting the analysis to the 22 elite lines the relationship between yield and thousand grain weight was completely removed (Fig. 4b) and an incipient linear trend, though not statistically significant, with grain number became apparent. This was mainly because the actual significant relationship was quadratic (r = 0.527, P < 0.01), implying that within this population of 22 elite hexaploid lines yield tended to increase with increases of grain number until intermediate values of this component and further increases in grain number tended to reduce yield (Fig. 4a). Essentially it could be seen that within the CIMCOG panel yield differences between genotypes were determined by particular combinations of grain number and grain weight of the different genotypes and then yield was not strongly related to any particularly numerical component (Fig. 4). There was a clear negative relationship between these major yield components (Fig. 5a). This negative relationship was stronger when considering the 22 elite lines than when the whole subset was taken into account (Fig. 5a). Due to the quadratic relationship between yield and grain number within the 22 elite lines (Fig. 4c) data-points crossed over the curves representing iso-yields at intermediate values of grain number: if compared with the lines with the lowest number of grains, the cultivars displaying intermediate values had smaller grains but not small enough to compensate for the increase in grain number, while, when genotypes increased grain number further the reduction in grain size was more than compensating the increase in grain number (Fig. 5a).

Fruiting efficiency was the trait most strongly explaining both yield components: the relationship was positive with grain number (Fig. 5b) and negative with grain weight (Fig. 5c), which would be the functional cause of the partial compensation between both yield components (Fig. 5a). The relationships mentioned between yield components and fruiting efficiency held for both the whole subset of 27 genotypes and for the analysis restricted to the 22 elite hexaploid lines (Fig. 5b,c), but they were stronger when restricting the analysis to the 22 elite hexaploid lines. Although there seemed to be an outlier in which fruiting efficiency was distinctly higher than for the rest of the population, the correlations coefficients would have been still significant if the analysis were made disregarding that particular genotype, particularly so for the analysis restricted to the 22 elite hexaploid lines (as after excluding that genotype of highest fruiting efficiency the correlation coefficients between fruiting efficiency and either grain number [r = +0.77_27lines_ P < 0.001 and +0.77_22lines_ P < 0.001] or grain weight [r = −0.59_27lines_ P < 0.001 and −0.76_22lines_ P < 0.001] remained highly significant).

### 3.4 Duration of phases and yield components

**Fig. 6:**
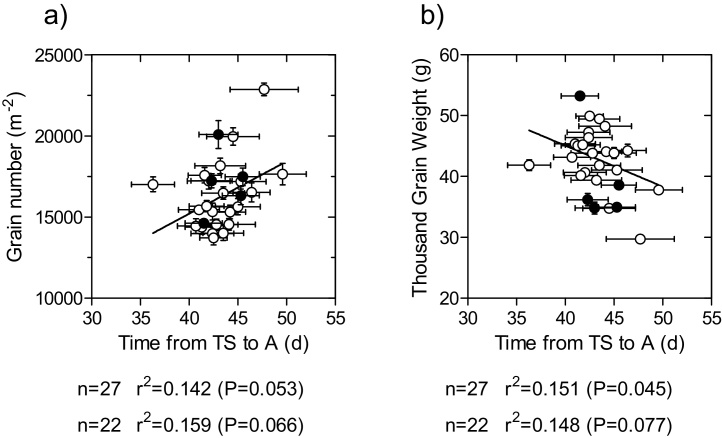
Relationships between either the number of grains per unit land area (a) or the average weight of grains estimated as thousand grain weight (b) and the duration of the late reproductive phase from terminal spikelet (TS) to anthesis (A). Each data-point is the average across the 4 environments and segments stand for the standard error of the means (not seen when smaller than the sizer of the symbol). Open circles represent the 22 elite hexaploid lines and closed circles represent the 4 historical and the *T. durum* cultivars.

The duration of the late reproductive phase tended to be related positively with the number of grains per unit land area (Fig. 6a) and negatively with the average weight of the grains (Fig. 6b). The relationships were similar when considering the whole subset or only the 22 elite genotypes. But in all cases the relationships were rather weak.

In the case of the relationship between grain weight and duration of the late reproductive phase (Fig. 6b) the fact that the length of the period from terminal spikelet to anthesis was the main determinant of time to anthesis (see above, and Fig. 3) could bring about the interpretation that the longer the late reproductive phase the later the grain filling condition and the smaller the grains. However, this explanation would be hardly plausible as the duration of the period from anthesis to maturity was very similar among all lines (see above and Fig. 2); and differences in thousand grain weight were chiefly determined by differences in the rate of grain filling (r = 0.99_27lines_ P < 0.001 and 0.98_22lines_ P < 0.001).

Regarding the weakness of the relationship between grain number and duration of the late reproductive phase (Fig. 6a), it implies that the main driving force for the genotypic differences in grain number was not the differences in spike dry weight at anthesis (the correlation between grain number and non-grain spike dry weight at 7 days after anthesis was extremely low; r = −0.09_27lines_ P = 0.62 and −0.17_22lines_ P = 0.45). As the difference in grain number among lines was largely explained by their differences in fruiting efficiency (Fig. 5b) there might be room for a subtle effect of the duration of the late reproductive phase on fruiting efficiency.

**Fig. 7:**
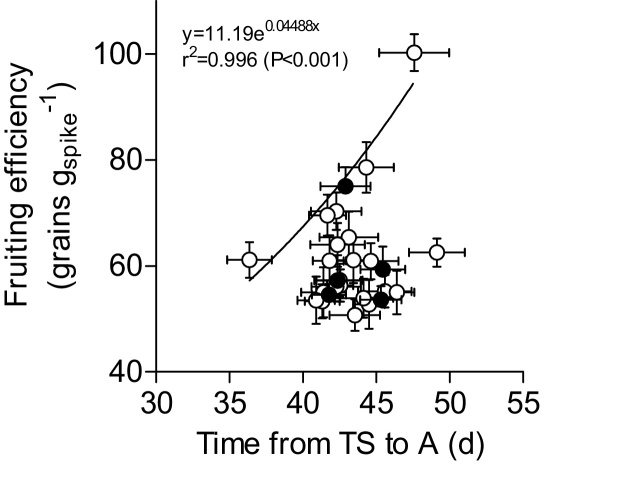
Relationships between fruiting efficiency and the duration of the late reproductive phase from terminal spikelet (TS) to anthesis (A). The solid line shows the boundary function (the equation and coefficient of determination were also included). Open circles represent the 22 elite hexaploid lines and closed circles represent the 4 historical and the *T. durum* cultivars. Each data-point is the average across the 4 environments and segments stand for the standard error of the means. See Table A.1 for more information in the genotype identification.

Analysing the relationship between fruiting efficiency and the length of the late reproductive phase produced a positive, though not significant, trend (Fig. 7). As the likely effect would be subtle it was not expected to find a highly significant degree of association between them. When analysing the relationship with a boundary function there was a rather strong positive relationship both for the whole subset and for the 22 elite hexaploid genotypes (Fig. 7), implying that the length of the late reproductive phase might set an upper threshold for fruiting efficiency.

### 3.5 Overall relationships through principal component analysis

**Fig. 8:**
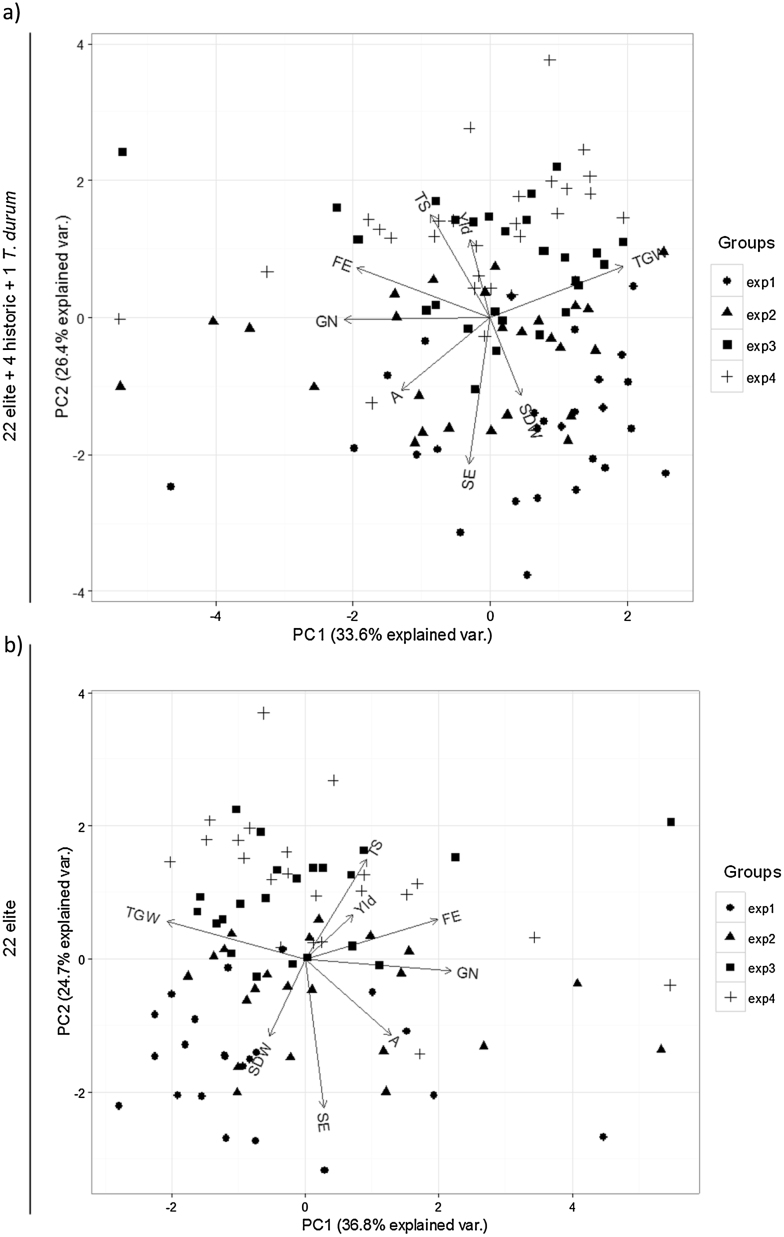
Biplot of principal components analysis considering the whole subset of the 27 genotypes (a) or only the 22 elite hexaploid genotypes (b) grown across 4 experiments (Table 1). Variables considered were Yld: grain yield, TGW: thousand grain weight, GN: grains per square meter, SDW: non-grain spike dry weight at 7 d after anthesis, TS: days from emergence to terminal spikelet, A: days from emergence to anthesis, SE: stem elongation period (days from TS to A), FE: fruiting efficiency.

The principal component analysis showed a greater variation of the yield determinants than yield itself, providing evidence that current elite material reach high yields by different sets of yield components. The variation across the four experiments is fairly captured, in both the whole 27 genotypes and 22 elite genotypes, by the two dimensions obtained from the analysis. Differences in yield considering the whole subset of 27 genotypes were virtually unrelated to increases in either grain number or grain weight (Fig. 8a). On the other hand, when analysing the subset of 22 elite hexaploid genotypes the scenario changes dramatically: yield seemed positively related to grain number per unit land area, while it was negatively related to thousand grain weight (Fig. 8b). Thus, across the G × E interaction for the analysis of the 22 elite hexaploid lines, the highest yielding genotypes were those able to increase grain number, even though there was a partial compensation in the average weight of grains.

In the biplots of the whole subset as well as in that of the 22 elite hexaploid lines there was a clear positive relationship between grain number and fruiting efficiency (and no relationship with spike dry weight at anthesis) and a strong negative relationship between fruiting efficiency and grain weight (Fig. 8a,b). It seemed that the main attribute responsible for major differences in grain number was in turn responsible for the grains set to be smaller.

## 4 Discussion

Native trait variation is key to further improvements independent of the use of GMO technologies. As breeders pyramid yield genes the most accessible variation is present within elite materials. Although searching for genetic variation in modern elite cultivars might be considered as ‘looking for the needle in the haystack’ (Able et al., 2007), several studies are far more enthusiastic suggesting that the genetic diversity within elite lines may still provide useful tools towards yield potential (Soleimani et al., 2002, Dreisigacker et al., 2004).

In the present study not only was there variation in the duration of phenological phases but also their durations seemed to be quite independent of each other. This was in agreement with studies carried out with other populations (Halloran and Pennell, 1982, Miralles and Richards, 2000, González et al., 2002, Whitechurch et al., 2007) Even though the CIMCOG is a panel selected mainly of elite material (i.e. well adapted and high-yielding), the wealth of variation within the panel is not surprising given that (i) CIMMYT germplasm is typically highly diverse with pedigrees incorporating landraces and products of interspecific variation including synthetics, and (ii) breeding programs generally do not assess or deliberately select for detailed phenology beyond heading and maturity date. Collectively the results support the idea of fine-tuning the developmental phases as a tool for improving not only adaptation but also yield potential (Slafer et al., 2001, Miralles and Slafer, 2007).

The lack of strong correlations between yield and yield components, imply that among the 27 genotypes, as well as for the 22 elite genotypes, there is more than one way to reach a high yield. Some high yielding genotypes had high grain number (Gonzalez-Navarro et al., 2015) while others have high grain weight (Quintero et al., 2014). Besides this, further improvements must be focused on grain number (Foulkes et al., 2011) as the plasticity of grain number is much larger than that of grain weight (Sadras and Slafer, 2012) and consequently any large increase in yield must require improvements in grain number (Slafer et al., 2014).

An increased stem elongation period could provide further allocation of biomass to the spike (i.e. a greater spike dry weight) at anthesis (Slafer et al., 2001, González et al., 2005b, Miralles and Slafer, 2007, González et al., 2011b). By providing more photo-assimilates to the spike through an extended stem elongation period, there could be an improvement in floret primordia survival (Ferrante et al., 2013) consequently increasing the number of fertile florets. However, making crosses for this purpose using the elite lines in the current study might be risky as there was no relationship between the length of the stem elongation phase and spike dry weight at anthesis. This means that lines possessing longer stem elongation phases in this panel may have also possess lower rates of canopy growth and/or lower levels of dry matter partitioning to the juvenile spikes compensating the expected advantage of longer late reproductive phase on spike dry weight at anthesis.

On the other hand, there was a subtle relationship between the duration of the late reproductive phase and fruiting efficiency, which is relevant, as the latter had a strong correlation with grain number. This supports the idea of using fruiting efficiency as an alternative trait to further increase grain yield (Slafer et al., 2015). In part, the relationship was only subtle because of the unexpected variation within the panel on time to anthesis. It would be likely that in another panel–varying less in time to anthesis- differences in duration of stem elongation phase may be more evident. At least this has been proven for individual genotypes when the duration of their stem elongation phase were modified artificially (Stockman et al., 1983, Savin and Slafer, 1991). Even though both fruiting efficiency and grain number had a highly significant negative correlation with grain weight, fruiting efficiency is shown to have a weaker association to grain weight than grain number. Similar results from González et al. (2014) provide some reassurance on using fruiting efficiency as a tool for the potential improvement of grain yield; notwithstanding potential drawbacks (Slafer et al., 2015). However, it is also true that the negative relationship between grain weight and fruiting efficiency may well represent a trade-off. Depending on the nature of the negative relationship, it might make improvements in fruiting efficiency either relevant or of little value to improve yield. Although data available from the present study does not allow us to elaborate further on the likely reason for the negative relationship, results from other studies suggest that the decrease in the average grain weight in high fruiting efficiency cultivars does not imply a constitutive effect on all grains (Ferrante et al., 2015, Elía et al., 2016), but rather evince the likely increased presence of more distal grains of smaller size potential reducing the average weight (Acreche and Slafer, 2006, Ferrante et al., 2015).

The relationship between the duration of stem elongation and fruiting efficiency was analysed with a boundary approach, which has been successfully used in other studies of complex traits (French and Schultz, 1984a, French and Schultz, 1984b, Sadras and Angus, 2006, Hunt and Kirkegaard, 2012a, Hunt and Kirkegaard, 2012b, Sadras and McDonald, 2012). Although unfeasible for direct selection in breeding programs due to the complexity of manipulating phenological phases, there is an increased use of marker assisted selection techniques that allow breeding programs to incorporate further improvement from complex traits (e.g. fruiting efficiency) to current elite lines (Dreisigacker et al., 2016). The analysis of the relationship showed that within this set of elite lines, traits other than duration of stem elongation phase were determining fruiting efficiency, but that the maximum possible value of fruiting efficiency would be only achievable with relatively long periods of stem elongation.

## Figures and Tables

**Table A.1:** Genotype identification (GID), year of release, cross name, and pedigree from the subset of 27 CIMCOG genotypes. In bold are the 4 historic lines and 1*T. durum.*

GID	Year	Cross Name	Pedigree
775	1966	SIETE CERROS T66 (historic)	PJ62/GB55
2465	1976	PAVON F 76 (historic)	VCM//CNO67/7C/3/KAL/BB
3895	1982	SERI M 82 (historic)	KVZ/BUHO//KAL/BB
16122	1988	BACANORA T 88 (historic)	JUP/BJY//URES
4556647	2002	MILAN/KAUZ//PRINIA/3/BAV92	MILAN/KAUZ//PRINIA/3/BAV92
5077000	2005	CIRNO C 2008 (*T. durum*)	SOOTY_9/RASCON_37//CAMAYO
6176346	2005	WBLL1*2/KIRITATI (BECARD)	WBLL1*2/KIRITATI
5343246	2005	CROC_1/AE·SQUARROSA (205)//BORL95/3/PRL/SARA//TSI/VEE#5/4/FRET2	CROC_1/AE·SQUARROSA (205)//BORL95/3/PRL/SARA//TSI/VEE#5/4/FRET2
5390612	2005	SUPER 152	PFAU/SERI.1B//AMAD/3/WAXWING
5397958	2005	BRBT1*2/KIRITATI	BRBT1*2/KIRITATI
5423688	2006	TC870344/GUI//TEMPORALERA M 87/AGR/3/2*WBLL1	TC870344/GUI//TEMPORALERA M 87/AGR/3/2*WBLL1
5995410	2008	TRAP#1/BOW/3/VEE/PJN//2*TUI/4/BAV92/RAYON/5/KACHU #1	TRAP#1/BOW/3/VEE/PJN//2*TUI/4/BAV92/RAYON/5/KACHU #1
5999777	2008	BABAX/LR42//BABAX/3/VORB	BABAX/LR42//BABAX/3/VORB
6000921	2008	SOKOLL//PBW343*2/KUKUNA/3/NAVJ07	SOKOLL//PBW343*2/KUKUNA/3/NAVJ07
6056245	2008	BCN/RIALTO	BCN/RIALTO
6171893	2009	CMH79A.955/4/AGA/3/4*SN64/CNO67//INIA66/5/NAC/6/RIALTO	CMH79A.955/4/AGA/3/4*SN64/CNO67//INIA66/5/NAC/6/RIALTO
6174886	2009	BECARD/KACHU	BECARD/5/KAUZ//ALTAR 84/AOS/3/MILAN/KAUZ/4/HUITES
6175024	2009	TACUPETO F2001/BRAMBLING*2//KACHU	TACUPETO F2001/BRAMBLING*2//KACHU
6175172	2009	YAV_3/SCO//JO69/CRA/3/YAV79/4/AE·SQUARROSA (498)/5/LINE 1073/6/KAUZ*2/4/CAR//KAL/BB/3/NAC/5/KAUZ/7/KRONSTAD F2004/8/KAUZ/PASTOR//PBW343	YAV_3/SCO//JO69/CRA/3/YAV79/4/AE·SQUARROSA (498)/5/LINE 1073/6/KAUZ*2/4/CAR//KAL/BB/3/NAC/5/KAUZ/7/KRONSTAD F2004/8/KAUZ/PASTOR//PBW343
6176178	2009	UP2338*2/4/SNI/TRAP#1/3/KAUZ*2/TRAP//KAUZ/5/MILAN/KAUZ//CHIL/CHUM18/6/UP2338*2/4/SNI/TRAP#1/3/KAUZ*2/TRAP//KAUZ	UP2338*2/4/SNI/TRAP#1/3/KAUZ*2/TRAP//KAUZ/5/MILAN/KAUZ//CHIL/CHUM18/6/UP2338*2/4/SNI/TRAP#1/3/KAUZ*2/TRAP//KAUZ
6176346	2009	WBLL1*2/KURUKU*2/5/REH/HARE//2*BCN/3/CROC_1/AE·SQUARROSA (213)//PGO/4/HUITES	WBLL1*2/KURUKU*2/5/REH/HARE//2*BCN/3/CROC_1/AE·SQUARROSA (213)//PGO/4/HUITES
6176523	2009	SAUAL/4/CROC_1/AE·SQUARROSA (205)//KAUZ/3/ATTILA/5/SAUAL	SAUAL/4/CROC_1/AE·SQUARROSA (205)//KAUZ/3/ATTILA/5/SAUAL
6177599	2009	KINGBIRD #1//INQALAB 91*2/TUKURU	KINGBIRD #1//INQALAB 91*2/TUKURU
6178401	2009	CNO79//PF70354/MUS/3/PASTOR/4/BAV92*2/5/FH6-1–7	CNO79//PF70354/MUS/3/PASTOR/4/BAV92*2/5/FH6-1–7
6178783	2009	SAUAL/WHEAR//SAUAL	SAUAL/WHEAR//SAUAL
6179128	2009	TACUPETO F2001/SAUAL//BLOUK #1	TACUPETO F2001/SAUAL//BLOUK #1
6179222	2009	PBW343*2/KUKUNA*2//FRTL/PIFED	PBW343*2/KUKUNA*2//FRTL/PIFED

**Table 1:** Description of environment, sowing, field trial setup, and meteorological data for the four experiments. Environment (4 experiments sowed in three years under irrigated conditions), Sowing (date of sowing and seed density), Plot size (long, wide, and setup of the plots), Available water (millimetres of rain throughout the crop cycle), Average temperature (mean daily temperature for the period between emergence to anthesis (E-A), and anthesis to maturity (A-M)), and average daily radiation (mean solar radiation).

Environment		Sowing	Plot size	Available water	Average temperature (°C)		Average daily radiation (MJ m^−2^ d^−1^)
					E-A	A-M	
Exp.1 Raised beds		06 Dec 2010 101 kg_seeds_ ha^−1^	5 m long and 4.16 m wide (4 raised beds 0.80 m wide, with 2 rows per bed, 0.24 m apart)	573 mm	14.9	19.7	21.8
Exp.2 flat beds		06 Dec 2010 101 kg_seeds_ ha^−1^	5 m long and 6 m wide (8 rows, 0.2 m apart)	573 mm	14.9	19.7	21.8
Exp.3 raised beds		09 Dec 2011 108 kg_seeds_ ha^−1^	8.5 m long and 2.08 m wide (3 raised beds 0.80 m wide, with 2 rows per bed, 0.24 m apart)	592 mm	15.2	19.3	21.6
Exp.4 raised beds		25 Nov 2012 110 kg_seeds_ ha^−1^	8.5 m long and 2.08 m wide (3 raised beds 0.80 m wide, with 2 rows per bed, 0.24 m apart)	600 mm	15.2	18.4	19.5

**Table 2:** Means, least significant difference (LSD α=0.05), coefficient of variation (CV), and mean squares of genotype (G) by environment (E) interaction (GxE) for yield components and main phenological traits of subset of 27 lines and four experiments.

Source of variation	Trait						
	Yld	GN	TGW	TS	SE	FE	SDW
Mean squares							
Environment	69632	72364612	106.89	1149.44	1539.8	1925.77	53545
Genotype	17015	50152422	320.18	47.97	64.23	1207.44	9113
GxE interaction	2669	2620653	5.73	8.76	16.87	144.05	6471
Residual	1356	1353401	3.95	2.94	4.71	134	7948

F-values							
Environment	51.35***	53.47***	27.06***	390.97**	326.92***	14.37***	6.74***
Genotype	12.55***	37.06***	81.06***	16.32***	13.64***	9.01***	1.15^ns^
GxE Interaction	1.97***	1.94***	1.45*	2.98***	3.58***	1.08^ns^	0.81^ns^

Yld: grain yield (g m^−2^), GN grains (m^−2^), TGW: thousand grain weight (g), TS: days from emergence to terminal spikelet, SE: stem elongation period (days from TS to anthesis), FE: fruiting efficiency (grains g_spike_^−1^), SDWa: non-grain spike dry weight at anthesis.

Significance: *** P < 0.001, ** P < 0.01, * P < 0.05, and ns not significant.
